# Quantitative proteomic analysis of the lysine acetylome reveals diverse SIRT2 substrates

**DOI:** 10.1038/s41598-022-06793-5

**Published:** 2022-03-09

**Authors:** Hui Zhang, Eric B. Dammer, Duc M. Duong, Diana Danelia, Nicholas T. Seyfried, David S. Yu

**Affiliations:** 1 Department of Radiation Oncology and Winship Cancer Institute, Emory University School of Medicine, 1365 Clifton Rd NE, C3008, Atlanta, GA 30322 USA; 2 Department of Biochemistry and Winship Cancer Institute, Emory University School of Medicine, 4133 Rollins Research Building, Atlanta, GA 30322 USA

**Keywords:** Cancer, Colorectal cancer, Biological techniques, Mass spectrometry, Proteomic analysis, Biochemistry, Protein-protein interaction networks

## Abstract

Sirtuin 2 (SIRT2) is a NAD+-dependent deacetylase, which regulates multiple biological processes, including genome maintenance, aging, tumor suppression, and metabolism. While a number of substrates involved in these processes have been identified, the global landscape of the SIRT2 acetylome remains unclear. Using a label-free quantitative proteomic approach following enrichment for acetylated peptides from SIRT2-depleted and SIRT2-overexpressing HCT116 human colorectal cancer cells, we identified a total of 2,846 unique acetylation sites from 1414 proteins. 896 sites from 610 proteins showed a > 1.5-fold increase in acetylation with SIRT2 knockdown, and 509 sites from 361 proteins showed a > 1.5-fold decrease in acetylation with SIRT2 overexpression, with 184 proteins meeting both criteria. Sequence motif analyses identified several site-specific consensus sequence motifs preferentially recognized by SIRT2, most commonly KxxxxK(ac). Gene Ontology, KEGG, and MetaCore pathway analyses identified SIRT2 substrates involved in diverse pathways, including carbon metabolism, glycolysis, spliceosome, RNA transport, RNA binding, transcription, DNA damage response, the cell cycle, and colorectal cancer. Collectively, our findings expand on the number of known acetylation sites, substrates, and cellular pathways targeted by SIRT2, providing support for SIRT2 in regulating networks of proteins in diverse pathways and opening new avenues of investigation into SIRT2 function.

## Introduction

Lysine acetylation is a common, dynamic, reversible, and evolutionarily conserved post-translational modification (PTM), important for regulating a number of protein functions, including interaction with binding partners, localization, catalytic activity, gene expression, conformation, and stability^1,2^. Lysine acetylation is regulated by acetyltransferases, which transfer acetyl groups from acetyl-coenzyme A (acetyl-CoA) to lysines, and deacetylases, which remove acetyl modifications. While lysine acetylation was initially discovered on histones^3^, it has since been shown to be a common PTM also on non-histone proteins^1,2^. Recent advances in quantitative mass spectrometry have vastly expanded the number of known proteins modified by acetylation, supporting the ubiquity of acetylation as a PTM^4^.

Sirtuin 2 (SIRT2) is a member of the sirtuin family of NAD+ dependent deacetylases, which regulate multiple biological processes, including genome maintenance, aging, tumorigenesis, and metabolism^5–9^. Significantly, mice deficient in *Sirt2* develop breast, liver, and other cancers^10,11^, suggesting that SIRT2 functions in tumor suppression. However, SIRT2 has also been paradoxically reported to have an oncogenic role or both oncogenic and tumor suppressive roles in other cancer types, including colorectal cancer^9,12,13^. Mice deficient in *Sirt2* also develop aging-related phenotypes, including neurological dysfunction^14,15^, cardiac dysfunction^16^, and arthritis^17^, as well as being protected against bacterial infections^18,19^, inflammation^20^, and neurodegeneration^21^; and reduced hepatic and renal injury^22,23^. Consistent with Sirt2’s in vivo role in multiple physiological conditions and disease states, SIRT2 has been reported to deacetylate a number of substrates involved in diverse biological processes, including genome maintenance, aging, myelination, mitosis, cellular differentiation, oxidative stress, cellular homeostasis, infection, inflammation, and autophagy^8,24–29^. While an ever-expanding number of SIRT2 substrates and deacetylation sites have been discovered—often in isolation, and the acetylomes of SIRT1, SIRT3, and SIRT7 have been reported^30–32^, the global landscape of the SIRT2 acetylome is less clear.

Here, using a label-free quantitative proteomic approach following enrichment for acetylated peptides from SIRT2-depleted and SIRT2-overexpressing HCT116 human colorectal cancer cells, we identified a total of 2846 unique acetylation sites within 1404 protein-coding gene products and 1414 protein isoforms. 896 acetylation sites from 610 proteins showed a > 1.5-fold increase in acetylation with SIRT2 knockdown, and 509 acetylation sites from 361 proteins showed a > 1.5-fold decrease in acetylation with SIRT2 overexpression with 184 proteins meeting both criteria, indicating a high likelihood of their being regulated by SIRT2 deacetylation. Sequence motif analyses identified several consensus acetylation site sequence motifs preferentially recognized by SIRT2. We further performed an array of bioinformatic analyses to categorize SIRT2 substrates into diverse pathways. Our findings expand on the number of known acetylation sites, substrates, and cellular pathways that are targeted by SIRT2, providing support for SIRT2 in regulating networks of proteins in diverse pathways.

## Results

### Proteome-wide identification of lysine acetylation changes following SIRT2 knockdown and overexpression

**Figure 1:**
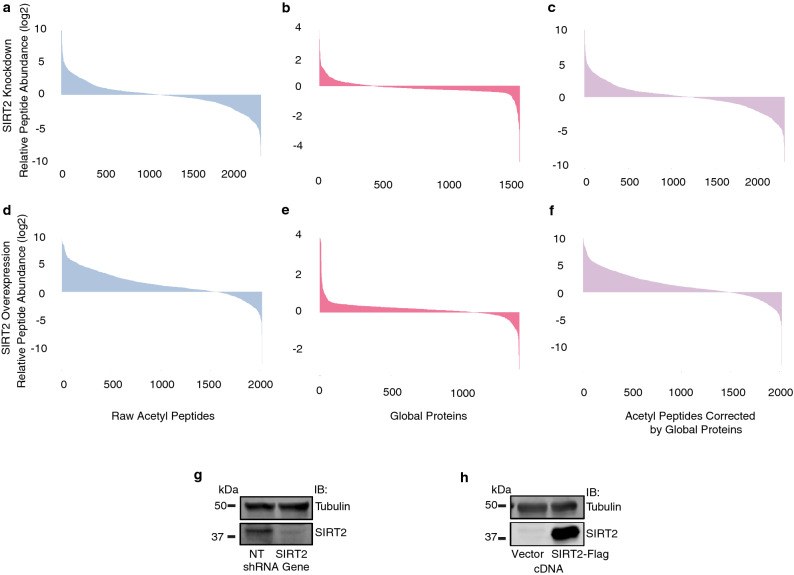
Proteome-wide identification of lysine acetylation following SIRT2 knockdown and overexpression. (**a**–**f**) represent respectively relative peptide abundance of acetyl peptides, global proteins, and acetyl peptides corrected by global proteins with SIRT2 knockdown (**a**–**c**) or overexpression (**d**–**e**). (**g**–**h**) Representative western blot analysis showing SIRT2 knockdown (**g**) or overexpression (**h**).

To identify the global landscape of the SIRT2 acetylome, we performed label-free quantitative proteomic analysis following enrichment for acetylated peptides using an anti-acetyl lysine antibody from SIRT2-depleted and SIRT2-overexpressing HCT116 human colorectal cancer cells using liquid chromatography-tandem mass spectrometry (LC–MS/MS) (Fig. 1a–f). Using a false discovery rate (FDR) of < 1%, we identified a total of 2846 unique lysine acetylation sites from 1414 protein isoforms of 1404 gene products (Supplementary Table S1). As we observed both changes in raw acetyl lysine peptide and global protein levels following SIRT2 knockdown and overexpression, to more accurately measure changes in acetylation levels of specific proteins, we normalized changes in the raw acetyl lysine peptide levels to the changes in their respective protein levels (Fig. 1c,f and Supplementary Fig. S1a,b). Western blot analysis confirmed SIRT2 knockdown and overexpression in HCT116 cells (Fig. 1g–h).

With a threshold of fold change > 1.5, SIRT2 knockdown induced 896 up-regulated lysine acetylation sites within 835 acetylated peptides in 610 unique proteins and 849 down-regulated lysine acetylation sites of 713 acetylated peptides in 458 unique proteins compared with a non-targeting (NT) siRNA control, whereas SIRT2 overexpression induced 1536 up-regulated lysine acetylation sites within 1398 acetylated peptides in 899 unique proteins and 509 down-regulated lysine acetylation sites within 450 acetylated peptides in 361 unique proteins compared with overexpression of an empty vector.

To obtain an overview of the acetylated proteins after SIRT2 manipulation, we conducted a gene ontology (GO)-Elite functional analysis^32^ on all identified proteins’ gene symbols (with a 1.5-fold change cutoff) based on their classification into biological processes, molecular functions, and subcellular localization GO categories. Supplementary Fig. S2 shows the GO classifications of the acetylated proteins enriched in changed lists after SIRT2 knockdown with Supplementary Fig. S2a indicating proteins with a decrease in acetylation and Supplementary Fig. S2b listing those with an increase in acetylation. Similarly, Supplementary Fig. S3 shows the GO classification of the acetylated proteins after SIRT2 overexpression, with Supplementary Fig. S3a indicating proteins with a decrease in acetylation and Supplementary Fig. S3b listing those with an increase in acetylation.

### Venn diagrams of acetylated lysine peptides and proteins reveal direct and indirect SIRT2 targets

**Figure 2:**
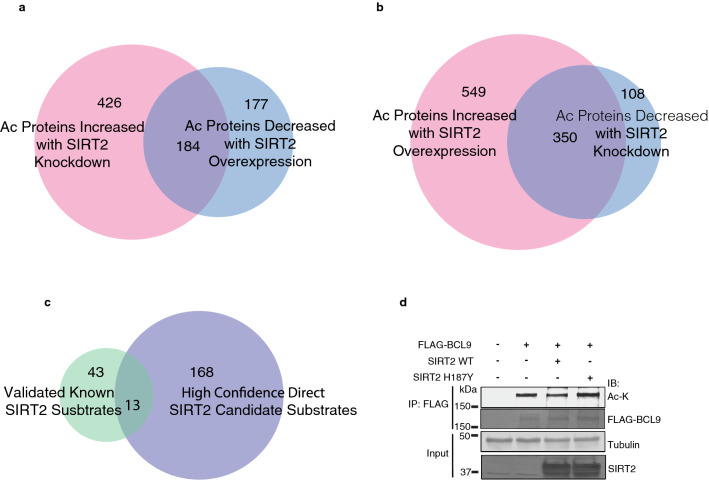
Venn diagrams of acetylated lysine proteins reveal direct and indirect SIRT2 targets. (**a**) Acetyl-lysine proteins upregulated 1.5 fold following SIRT2 knockdown and downregulated 1.5 fold following SIRT2 overexpression. (**b**) Acetyl-lysine proteins upregulated 1.5 fold following SIRT2 overexpression and downregulated 1.5 fold following SIRT2 knockdown. (**c**) Venn diagram of validated known SIRT2 substrates with high confidence direct SIRT2 substrates discovered in the present study. Same proteins with multiple gene symbols are consolidated into one. (**d**) 293 cells were transfected with FLAG-BCL9 together with histone acetyltransferases, and wild-type FLAG-SIRT2 or deacetylase-inactive FLAG-SIRT2 H187Y in the presence of TSA, immunoprecipitated with anti-FLAG agarose beads, separated by SDS-PAGE, and immunoblotted with antibodies against FLAG, acetyl-lysine, and α-Tubulin.

To identify high confidence SIRT2-directed downstream effectors, we further examined an opposing correlation of acetylation changes between SIRT2 knockdown and overexpression. Specifically, we generated Venn diagrams of acetylated lysine peptides, including ones with opposing changes in both SIRT2 knockdown and SIRT2 overexpression. For instance, Fig. S4a shows the correlation between up-regulated acetyl peptides in SIRT2 knockdown and down-regulated acetyl peptides in SIRT2 overexpression with identical corresponding peptides. And Fig. S4b shows the overlap between down-regulated acetyl peptides in SIRT2 knockdown and up-regulated acetyl peptides in SIRT2 overexpression. In addition, we also conducted opposing correlations of acetylation changes between SIRT2 knockdown and SIRT2 overexpression at the protein level independent of sites (Fig. 2). Figure 2a shows the protein level overlap of up-regulated hits in SIRT2 knockdown and down-regulated hits in SIRT2 overexpression, whereas Fig. 2b shows the overlap of down-regulated hits with SIRT2 knockdown and up-regulated hits with SIRT2 overexpression. We consider proteins with upregulation of acetylation after SIRT2 knockdown, or downregulation of acetylation after SIRT2 overexpression, as SIRT2 direct targets; whereas, proteins with downregulation of acetylation after SIRT2 knockdown, or upregulation of acetylation after SIRT2 overexpression, as SIRT2 indirect targets. The proteins or peptides in the overlapping part of the knockdown and overexpression areas of each Venn diagram would be considered high confidence SIRT2 targets (hits). 134 acetylated peptides with 152 lysine acetylation sites showed increased acetylation after SIRT2 knockdown and decreased acetylation after SIRT2 overexpression (Fig. S4a), suggesting that these acetylation sites are direct SIRT2 deacetylation targets. Interestingly, 419 acetylated peptides with 504 lysine acetylation sites were paradoxically found to increase in acetylation after SIRT2 overexpression and decrease in acetylation after SIRT2 knockdown (Fig. S4b), suggesting that their acetylation may be indirectly dependent on regulation downstream of SIRT2. In addition, we also correlated the hits at the protein level independent of lysine sites, and found 184 proteins as direct hits (Fig. 2a) and 350 proteins as indirect hits (Fig. 2b) respectively. To identify how many of the direct SIRT2 targets are validated known SIRT2 substrates, we conducted an extensive literature review to curate experimentally validated SIRT2 substrates (Supplementary Table S4). As a result, we were able to identify 56 experimentally validated known SIRT2 substrates. More importantly, we found that 13 proteins are among our high-confidence direct SIRT2 target list and the known SIRT2 substrates, accounting for 23% of the total known list (Fig. 2c and Supplementary Table S4). This finding demonstrates that our mass spectrometry analysis here can identify legitimate SIRT2 substrates, further supporting the validity and reliability of our study. Indeed, we found that FLAG-BCL9 is deacetylated by FLAG-SIRT2 WT but not catalytically inactive H187Y expressed in 293 cells (Fig. S2d), providing validation for BCL9 as a novel SIRT2 substrate.

### Gene ontology (GO)-Elite analysis of SIRT2 target proteins reveals diverse cellular processes and functions

**Figure 3:**
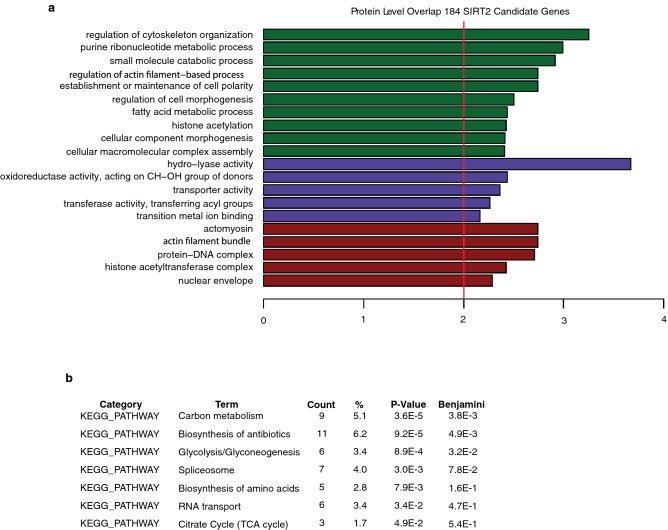
Gene Ontology (GO) Elite and KEGG pathway enrichment analysis of SIRT2 target proteins reveals diverse cellular processes and functions. (**a**, **b**) GO Elite classification and KEGG pathway enrichment analysis of acetylated proteins both upregulated 1.5-fold following SIRT2 knockdown and downregulated 1.5-fold following SIRT2 overexpression.

To determine if SIRT2 deacetylation targets may have a functional relationship, we further conducted serial in-depth enrichment analyses with the lists of opposing overlapping hits (high confidence hits). We first conducted GO-Elite analysis using the 184 protein level direct hits identified in Fig. 2a. GO-Elite analysis based on the results of the biological process category (Fig. 3a, green bars), suggests that SIRT2 functions in highly diverse biological processes. For instance, regulation of cytoskeleton organization, purine ribonucleotide metabolic process, small molecule catabolic processes, and regulation of cell morphogenesis, as well as histone acetylation were enriched terms associated with these acetylated proteins. In the molecular function category (Fig. 3a, blue bars), hydro-lyase activity, transporter activity, transferase activity (transferring acyl group), and transition metal ion binding were significantly enriched. Cellular components (Fig. 3a, red bars) enriched among these proteins included actomyosin, actin filament bundles, protein-DNA complex, histone acetyltransferase complex, and the nuclear envelope.

We also conducted a GO Elite analysis with the gene products represented among the 134 hits based on peptide level overlap from Fig. S4a shown in Supplementary Fig. S5a. In terms of cellular component category, the analysis demonstrated very similar enrichments compared to the protein hits list (Supplementary Fig. S5a and Fig. 3a, red bars), with both comprising terms for actomyosin, actin filament bundles, histone acetyltransferase complex, and protein-DNA complex. Several GO terms were also consistently observed in the biological process category as those in the protein list, including cellular component morphogenesis, regulation of cytoskeleton organization, purine ribonucleotide metabolic process, and histone acetylation (Supplementary Fig. S5a and Fig. 3a, green bars). With respect to the molecular function category, guanyl nucleotide binding was the only group enriched in acetylated peptides (Supplementary Fig. S5a, blue bars). Note that acetylation and acetyltransferase are recurring themes repeatedly shown among all three categories of protein hits GO analysis and two categories of peptide hits’ GO analysis. These results suggest that the present acetylome is representative of acetylation functionality as a significantly overrepresented category among the hit list, further indicating the reliability of our dataset. Additionally, we also conducted GO-Elite using the Gene Set Enrichment Analysis (GSEA) C2 molecular signatures database collection of curated canonical pathway gene sets and found a significant enrichment of tumor-related gene sets (Supplementary Fig. S5b).

### KEGG pathway analysis demonstrates key enrichments in metabolism and RNA regulation

**Figure 4:**
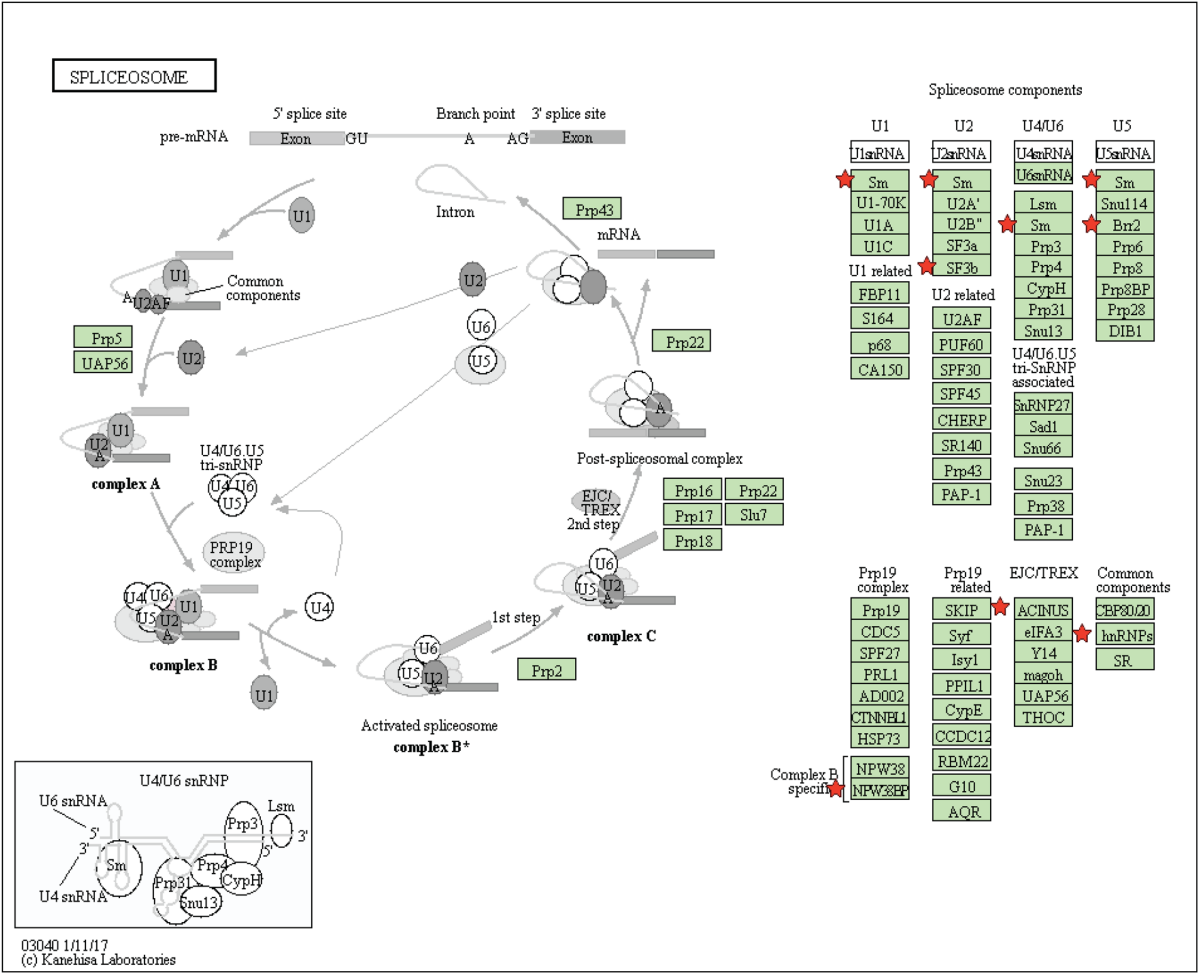
KEGG pathway enrichment analysis of the acetylated proteins in the spliceosome. KEGG pathway enrichment analysis of acetylated proteins both upregulated 1.5-fold following SIRT2 knockdown and downregulated 1.5-fold following SIRT2 overexpression that function in the spliceosome. Red stars indicate the hits from the present study.

To determine whether SIRT2 substrates function in specific pathways, we utilized the Kyoto Encyclopedia of Genes and Genomes (KEGG) pathway analysis^33^. Using a p-value < 0.05 as a cutoff, we identified seven significantly enriched KEGG pathways, including carbon metabolism, biosynthesis of antibodies, glycolysis/gluconeogenesis, spliceosome, biosynthesis of amino acids, RNA transport, and the citrate cycle (TCA cycle) (Fig. 3b). Metabolic pathways were highly represented with three pathways among these seven. Carbon metabolism was identified as the top-ranked KEGG pathway, with nine proteins in the list represented, including aldolase, fructose-bisphosphate A (ALDOA), dihydrolipoamide dehydrogenase (DLD), enolase 1 (ENO1), enoyl-CoA hydratase, and 3-hydroxyacyl CoA dehydrogenase (EHHADH), glyceraldehyde-3-phosphate dehydrogenase (GAPDH), hydroxyacyl-CoA dehydrogenase/3-ketoacyl-CoA thiolase/enoyl-CoA hydratase (trifunctional protein), alpha subunit (HADHA), malate dehydrogenase 2 (MDH2), the muscle-expressed pyruvate kinase gene product (PKM2), and triosephosphate isomerase 1 (TPI1). Glycolysis/gluconeogenesis and the TCA cycle were the other two highly represented KEGG pathways ranked 3rd and 7th, respectively with six and three genes from the list involved in these pathways. Interestingly, RNA function-related KEGG pathways were also highly represented: spliceosome and RNA transport. Specifically, there were seven genes in the list involved in the spliceosome, including PHD finger protein 5A (PHF5A), WW domain binding protein 11 (WBP11), apoptotic chromatin condensation inducer 1(ACIN1), heterogeneous nuclear ribonucleoprotein C (C1/C2) (HNRNPC), small nuclear ribonucleoprotein U5 subunit 200 (SNRNP200), heterogeneous nuclear ribonucleoprotein U (HNRNPU), and small nuclear ribonucleoprotein polypeptide G (SNRPG) (see detailed pathway map in Fig. 4). Separately, there were six genes in the list involved in RNA transport, including Ran GTPase activating protein 1 (RANGAP1), ACIN1, eukaryotic translation initiation factor 4 gamma 2 (EIF4G2), nucleoporin 153 (NUP153), nucleoporin 214 (NUP214), and nucleoporin 50 (NUP50) (see detailed pathway map in Fig. S6).

### Motifs and properties of SIRT2 regulated acetylated lysine peptides

**Figure 5:**
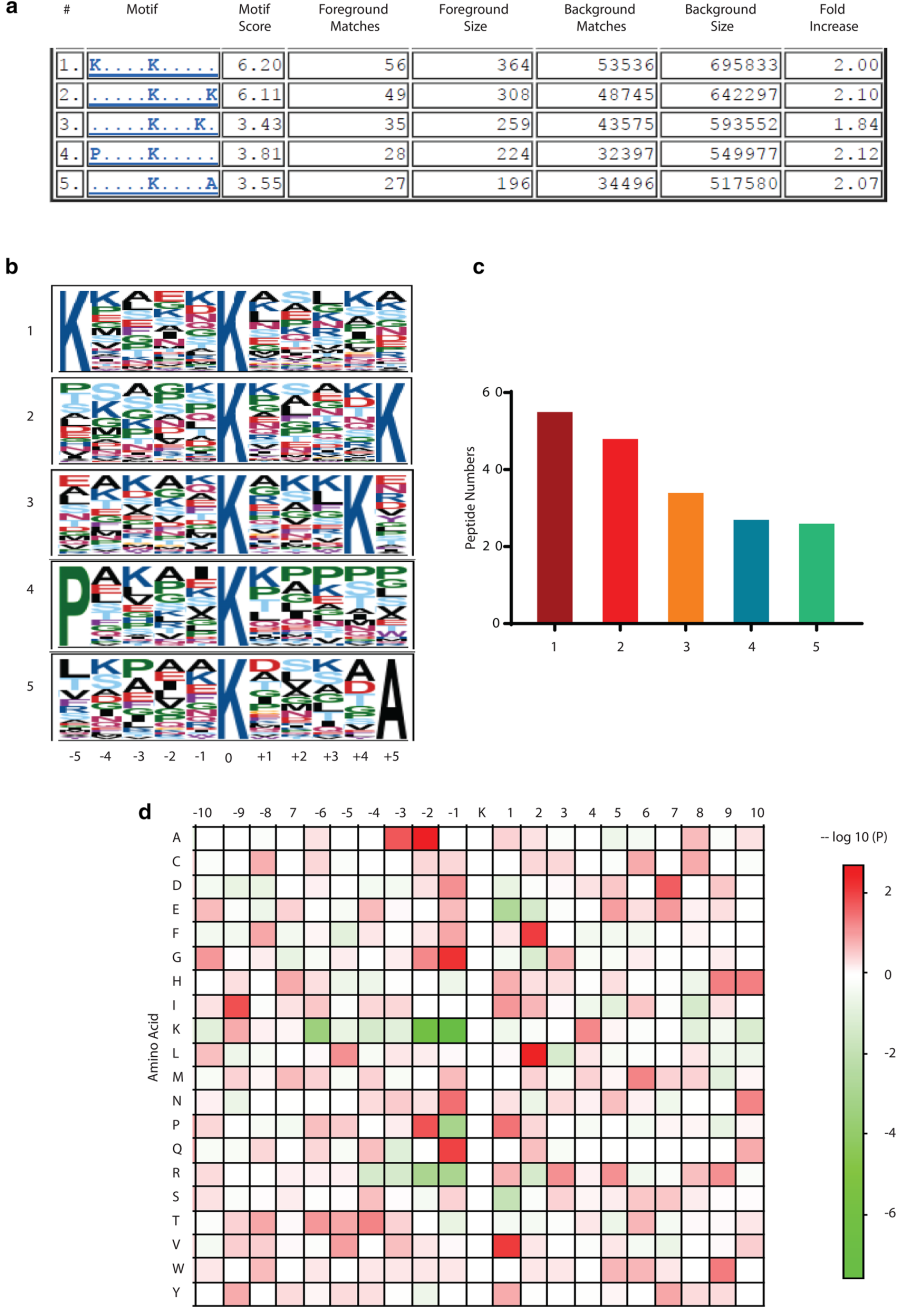
Properties of identified acetylated lysine peptides. (**a**,** b**) Acetylation motifs and conservation of acetylation sites identified by Motif-X. (**c**) Quantification of acetylation motifs identified by Motif-X. (**d**) Heat map of the amino acid compositions of the acetylated lysine sites showing the relative abundance of the adjacent 20 amino acids surrounding the acetylated lysine. The colors in the heat map represent the – log10 of P value (red shows over-representation, green shows under-representation).

Several lysine acetylation sequence motifs have been characterized^34–40^, and an in vitro screen of an acetylome microarray peptide library with all 7 sirtuins has suggested that sirtuins may have overlapping but varying sequence selectivity^41^; however, it is not known if SIRT2 may preferentially recognize certain acetylation site sequence motifs in vivo. Using the Motif-X program^42^, we extracted 5 enriched motifs, with amino acid sequences from − 5 to + 5 residues surrounding the acetylated lysine site(s) from 134 acetylated peptides (Fig. 5a–c). These motifs include: -KxxxxK(ac)-, -K(ac)xxxxK-, -K(ac)xxxK-, -PxxxxK(ac)-, and -K(ac)xxxxA- (Fig. 5a,b), where x is any other amino acid. -KxxxxK(ac)- was the most common motif, represented by 53 site(s) of the 134 acetylated peptides (Fig. 5c). In a complementary approach, we used the Multiple Expression Motifs for Motif Elicitation (MEME) algorithm^43^ to identify overrepresented motifs with a high frequency of lysine and found two conserved motifs by this method (Supplementary Fig. S7). We next analyzed the relative abundance of amino acid residues flanking the acetylation sites represented by an intensity map using MEME (Fig. 5d). Specifically, MEME was utilized to generate a peptide residue position frequency matrix for the 134 site-centered peptides with flanking residues ± 10 residues from the acetylation site, and then that matrix was used to perform a Fisher ‘s exact test, generating a − log_10_ (p-value) signed heatmap for each of the 20 amino acids across the site-centered 21-residue sequence window representing amino acid overuse in red and underuse in green at each position from − 10 to + 10 residues away from the center-positioned acetylated lysine residue. This is a heat map of the amino acid overrepresentation and underrepresentation significance relative to chance of residues flanking the acetylated sites. Almost all the aliphatic amino acids (nonpolar and hydrophobic) have a high representation near the flanking region of the acetylated lysine, suggesting a crucial role of hydrophobic interactions in SIRT2 recognition of targeted acetylated lysine residues. In particular, amino acids alanine (A) and glycine (G) have the strongest overrepresentation at the left side of the acetylated lysine, with A at − 2 and − 3 positions, and G at − 1 and − 2 positions. Conversely, valine (V) and leucine (L) have the strongest overrepresentation at the right side (+ 1 and + 2 positions, respectively). Proline, on the other hand, has modestly high overrepresentation on both sides of the acetylated lysine, (− 2 and + 1 positions). By contrast, all the alkaline amino acids (lysine, K; arginine, R; and histidine, H) are underrepresented immediately left of the acetylated lysine, (particularly at positions − 1 to − 4). In contrast, they have a modestly high frequency of occurrence C-terminal of the acetylated lysine, particularly at relatively distant positions (K at + 4; R at + 3, + 5, and + 9 positions and H at + 1, + 9, and + 10).

### MetaCore molecular function enrichment analysis and interaction networks

**Figure 6:**
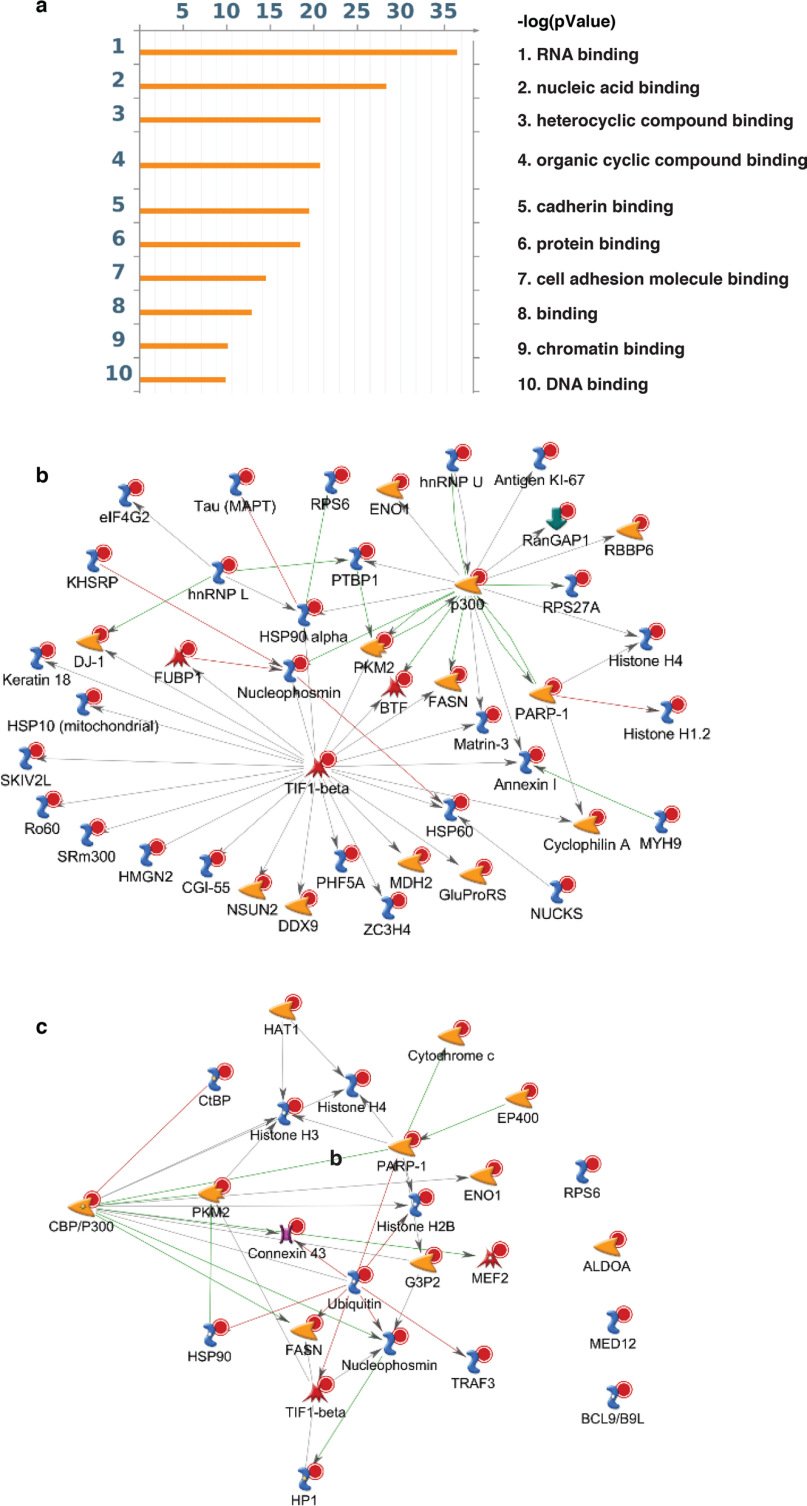
MetaCore molecular function enrichment analysis and interaction network. (**a**) MetaCore molecular function enrichment analysis of acetylated proteins both upregulated 1.5-fold following SIRT2 knockdown and downregulated 1.5-fold following SIRT2 overexpression. (**b**, **c**) Interaction network of acetylated proteins both upregulated 1.5-fold following SIRT2 knockdown and downregulated 1.5-fold following SIRT2 overexpression that function in RNA binding (**b**) and transcription regulation (**c**).

To identify relationships and connectivity among our SIRT2 targets, we conducted a molecular function enrichment analysis using the MetaCore bioinformatics platform (genego.com) (Fig. 6a). RNA binding was the most significantly enriched molecular function, represented by the largest group of proteins (85 out of 184 acetylated proteins), consistent with enrichment of the spliceosome and RNA transport in our KEGG pathway analysis. We therefore generated an interaction network using the MetaCore platform for RNA binding proteins. To define the most closely linked and central connections, we used the Direct Interaction algorithm, the most stringent algorithm, which allows the visualization of only direct connectivity between root nodes (i.e. proteins only from the provided list). As expected, a significant number of the proteins were not directly connected in this manner. After removing un-connected proteins, we obtained a pathway map with two centralized hubs: TIF1β and p300 (Fig. 6b). In addition to these two centralized hubs, there were also several proteins serving as secondary central nodes: HSP90α, PARP1, and nucleophosmin each have 5 interactions, while hnRNPL and PKM2 each have 4 interactions (Fig. 6b). Significantly, both p300 and PKM2 are known SIRT2 substrates^44,45^, supporting the feasibility of our approach and the validity of our dataset, and suggesting that p300 and PKM are key downstream effectors of SIRT2.

Transcription regulation, which is represented by 29 out of 184 acetylated proteins, was the top ranked pathway in a pathway map folder analysis using MetaCore (Fig. S8a). Three transcription-related networks were also enriched among the top 15 ranked MetaCore process networks (Supplementary Fig. S8b). A protein interaction network was generated from the 29 acetylated proteins involved in transcription regulation, which revealed ubiquitin, PARP1, and CBP/p300 as the most central hubs, and histone H3, TIF1β and, nucleophosmin as secondary hubs (Fig. 6c).

Consistent with SIRT2’s role in orchestrating the DNA damage response (DDR), the DDR, represented by 16 out of 184 acetylated proteins, was the second ranked pathway in pathway map folder analysis using MetaCore (Fig. S8a). A similar protein interaction network was generated from the 16 acetylated proteins involved in the DDR, which revealed PARP1 and p300 as the most central hubs, and histone H2B, CBP, and ubiquitin as secondary hubs (Fig. S9a). The cell cycle and its regulation, which are represented by 12 out of 184 acetylated proteins, was also a highly ranked pathway. (Fig. S8a). A protein interaction network revealed CBP/p300 and ubiquitin as the most central hubs (Fig. S9b). Finally, 10 tumor related pathways were enriched amongst the top 20 ranked pathways in the MetaCore pathway map folder analysis (Fig. S8a), suggesting that proteins important in tumorigenesis are key SIRT2 targets. We dually noted that, the above is consistent with our GO GSEA C2 custom database enrichment analysis (Fig. S5b), which discovered a significant enrichment of tumor-related GO terms. To explore the molecular basis of colorectal tumorigenesis specifically, we generated an interaction network with the proteins functioning in colorectal neoplasms among SIRT2 direct hits. As shown in Fig. S9c, an interaction network with these proteins revealed ubiquitin and p300 as two central hubs, with PARP1 and CBP being secondary hubs.

## Discussion

Our findings provide proteomic analysis of the global landscape of the SIRT2 acetylome with both loss-of-function and gain-of-function models, revealing previously unidentified acetylation sites, proteins, and pathways targeted by SIRT2. In this regard, we identified a total of 2,846 unique acetylation sites from 1414 proteins, with 896 acetylation sites from 610 proteins showing a > 1.5-fold increase in acetylation with SIRT2 knockdown, and 509 acetylation sites from 361 proteins showing a > 1.5-fold decrease in acetylation with SIRT2 overexpression and 134 identical acetyl peptides or 184 proteins meeting both criteria, expanding on the number of known acetylation sites and substrates targeted by SIRT2. We further performed a series of sequence motif analyses of the enriched acetylated peptides targeted by SIRT2, which identified several consensus acetylation site sequence motifs preferentially recognized by SIRT2 that will aid in the identification of additional novel SIRT2 deacetylation sites. In addition, Gene Ontology, KEGG, and MetaCore pathway analyses identified that SIRT2 substrates are involved in diverse pathways, such as carbon metabolism, glycolysis, the spliceosome, RNA transport, RNA binding, transcription, the DNA damage response, the cell cycle, and colorectal cancer, expanding on the cellular pathways targeted by SIRT2, thus providing support for SIRT2 in regulating networks of proteins in diverse pathways and opening new avenues of investigation into SIRT2 function.

168 of the 184 high confidence SIRT2 direct substrate proteins identified in our study have not previously been reported, revealing the depth of our proteomic analysis in identifying novel SIRT2 deacetylation sites. Indeed, using a cellular deacetylation assay, we validated one of these proteins, BCL9 as a novel SIRT2 substrate. We also identified a total of 2846 acetylation sites within 1414 proteins; 896 acetylation sites showed a > 1.5-fold increase in acetylation with SIRT2 knockdown, and 509 acetylation sites showed a > 1.5-fold decrease in acetylation with SIRT2 overexpression. Although many of these acetylation sites did not meet our stringent criteria of regulated acetylation with both SIRT2 knockdown and SIRT2 overexpression, they may still be legitimate SIRT2 deacetylation sites as lysine sites which are highly acetylated at baseline may not show a > 1.5-fold further increase in acetylation and lysine sites which are usually not acetylated or transiently acetylated at baseline may not show a > 1.5-fold further decrease in acetylation with SIRT2 overexpression. In addition, we do not expect our proteomic analysis to be fully saturating for detecting all sites of SIRT2-regulated acetylation due to the use of a stringent FDR of < 1%, and technical limitations such as inefficiencies in enrichment of acetylated peptides and insufficient sensitivity for detection of some poorly ionizing peptides by LC–MS/MC, and the lack of expression of all gene products in the specific conditions utilized, such as that of the cell type used for the source of acetylated peptides for enrichment.

It is interesting that we also identified 419 acetyl peptides or 350 proteins which showed paradoxically both a corresponding > 1.5-fold decrease in acetylation with SIRT2 knockdown and > 1.5-fold increase in acetylation with SIRT2 overexpression, suggesting negative regulation of acetylation by SIRT2. It has previously been reported that SIRT2 and the p300 acetyltransferase negatively regulate each other^44,46^. SIRT2 deacetylates p300, which impairs its autoacetylation^44^, while p300 acetylates SIRT2, which impairs its deacetylase activity^46^. While this negative regulation of p300 by SIRT2 would not fully explain SIRT2’s role in promoting acetylation, it may be possible that the decrease in acetylation observed with SIRT2 knockdown and increase in acetylation with SIRT2 overexpression may be attributed indirectly to SIRT2’s positive regulation of additional acetyltransferases, which then acetylates these SIRT2-regulated sites. Indeed, several other acetyltransferases, including CBP and HAT1, were also identified in our analysis as potential SIRT2-regulated substrates. We also discovered several acetylation-related proteins such as BRD1, EP400, and MEAF6. Both MEAF6 and EP400 are components of a histone acetyltransferase complex called NuA4, which functions to acetylate histones H4 and H2A^47^, thereby transcriptionally activating select genes. BRD1 is a subunit of the MOZ/MORF acetyltransferase complex and induces acetylation of histone H3^48^. All of these proteins could lead to acetylation of sites promoted by SIRT2. Given the significant number of lysine sites whose acetylation are paradoxically promoted by SIRT2, determining the mechanism by which SIRT2 may indirectly promote acetylation of these sites would be of interest for future investigation.

Our sequence motif analyses identified several consensus acetylation site sequence motifs preferentially recognized by SIRT2, most commonly -KxxxxK(ac)- but also -K(ac)xxxxK-, -K(ac)xxxK-, -PxxxxK(ac)-, and -K(ac)xxxxA-. Furthermore, we found a preference for A at the − 2 and − 3 positions, G at the − 1 and − 2 positions, V and L at the + 1 and + 2 positions respectively, P at the − 2 and + 1 positions, and alkaline amino acids (K, R, H) at relatively distant C-terminal (positive) positions. Consistent with our findings, SIRT2 has been reported to favor positively charged residues at + 4 and slightly on the amino-terminal side and disfavor negatively charged residues at the + positions in vitro^41^, although a more limited in silico analysis of published SIRT2 substrates found no clear consensus sequence for SIRT2^49^. Previous sequence motif analyses from acetylome LC–MS/MS studies have indicated that SIRT1 has a similar preference for A and G at the − 1 and − 2 positions and E at the + 2 position^30^; SIRT3 has a preference for a positive charge at the + 1 position or K at the + 1 and + 2 positions^31^; and no evidence for a preferred acetylation site sequence motif was observed for SIRT7^32^. Thus, our findings provide further support for the concept that sirtuins may have overlapping but varying sequence selectivity.

Our Gene Ontology, KEGG, and MetaCore pathway analyses identified SIRT2 substrates involved in diverse pathways, including carbon metabolism, glycolysis, the spliceosome, RNA transport, RNA binding, transcription, the DNA damage response, the cell cycle, and colorectal cancer. These data expand on the cellular pathways targeted by SIRT2, thus providing support for SIRT2 in regulating networks of proteins in diverse pathways and opening new avenues of investigation into SIRT2 function.

## Methods

### Cell culture

HCT116 cells were obtained from ATCC (CCL-247) and were grown in DMEM supplemented with 7.5% (vol/vol) FBS. Stable HCT116 cells were grown in 1ug/ml puromycin (Fisher).

### Transfections

Transfections were done on 5 million cells in 60 mm plates using Lipofectamine 2000 (Invitrogen) and performed per the manufacturer’s instructions. Cells were split after 16 h of incubation and allowed to recover for a further 48 h post-transfection before harvest. Importantly, for mass spectrometric analyses, overexpression of SIRT2 or control vector was concomitant with overexpression of histone acetyltransferases to offset potential decreases of the underlying stoichiometry in acetylation caused by SIRT2 overexpression.

### Immunoblot

Cells were harvested in PBS and lysed for 30 min on ice in Nonidet P-40 buffer (200 mM NaCl, 1% Nonidet P-40, 50 mM Tris·HCl pH 8.0) freshly supplemented with protease inhibitors. Lysates were clarified by centrifugation (15,700×*g*, 10 min at 4 °C), and the supernatants were then collected. Protein samples were then quantified with Bradford assay and resolved by SDS/PAGE, transferred onto PVDF, and probed using the appropriate primary antibodies. Membranes were trimmed sideways before hybridizing with antibodies to reduce costs by using less antibodies. The full length of the molecular weight range remains intact. Detection was performed with the Odyssey system. The antibodies used were as follows: SIRT2 (Santa Cruz; sc-20966), tubulin (Sigma; T6074).

### Cellular deacetylation assay

293 cells were transiently co-transfected with Flag-BCL9, together with histone acetyltransferases (P300/CBP/pCAF), along with FLAG-SIRT2-WT or FLAG-SIRT2-H187Y, and cultured with 0.5 μM TSA for 12 h. Cells were harvested in PBS and lysed for 20 min on ice in IP lysis buffer (0.75% CHAPS, 10% glycerol, 150 mM NaCl, 50 mM Tris pH 7.5) freshly supplemented with protease inhibitors and 1uM TSA. Lysates were clarified by centrifugation (13,000 rpm, 15 min at 4 °C), the supernatants were then collected and diluted by same volume of dilution buffer (10% glycerol, 150 mM NaCl, 50 mM Tris pH 7.5) to adjust the CHAPS concentration to 0.375%. Protein concentration was then determined and lysates of 2 mg protein were used for immunoprecipitation reaction, protein lysates were immunoprecipitated using anti-FLAG M2 agarose (Sigma). The immunocaptured proteins were analyzed for deacetylation by immunoblotting with anti-FLAG and anti-acetyl antibody. The antibodies used were as follows: tubulin (Sigma; T6074), FLAG (Santa Cruz, sc-51590), acetyl lysine (Cell Signaling, 9441).

### Cell and protein harvesting, and digestion into peptides for subsequent IP, LC–MS/MS

Cells were harvested by scraping of plates and centrifugation into a pellet at 2900×*g* (rcf). The cells were lysed and homogenized in 8 M urea buffer (8 M urea, 10 mM Tris, 100 mM NaH_2_PO4 buffer, pH 8.5, supplemented with HALT protease and phosphatase cocktail inhibitors (Thermo Fisher Scientific, # 78440) using a Bullet Blender (Next Advance) per manufacturer’s protocol. Each sample was placed in a 1.5 ml Rino tube containing 750 mg stainless steel beads (0.9–2 mm in diameter). Five hundred µl 8 M urea lysis buffer was added to each sample and blended twice for 5 min at 4 °C. Homogenates were transferred to clean Eppendorf tubes and centrifuged at 10,000×*g* for 5 min and sonicated (Sonic Dismembrator, Fisher Scientific) 3 times for 5 s with 15 s intervals of rest at 30% amplitude to disrupt nucleic acids. Protein concentration was determined by the bicinchoninic acid (BCA) method. Protein homogenates were diluted with 50 mM NH_4_HCO_3_ to approximately 2 M urea concentration and reduced using 1 mM 1,4-dithiothreitol (DTT) for 30 min and alkylated with 5 mM iodoacetamide (IAA) for 30 min in the dark. Proteins were digested with Lys-C (Wako; 1:100 enzyme: substrate ratio) at room temperature for 3 h followed by further overnight digestion with trypsin (Promega; 1:50 enzyme: substrate ratio) at room temperature. Tryptic peptides were subsequently acidified using 1% formic acid (FA) and 0.1% trifluoroacetic acid (TFA) before desalting and purification using Sep-Pak C18 columns (Waters) followed by peptide elution in 50% acetonitrile.

### Peptide immunoprecipitation with a pan-acetyl-lysine antibody

Peptide samples were enriched for acetyl-lysine using the Cell Signaling Technology PTMScan kit for acetyl-lysine (#13416) per the manufacturer’s protocol. Briefly, 5 mg of desalted, purified peptides were reconstituted in IAP buffer (Cell Signaling, #9993), followed by clearing via centrifugation at 10,000×*g* at 4 °C. PBS-washed antibody-bead slurry was combined with the peptides and incubated at 4 °C for 2 h with gentle rotation. Beads were washed 2 × with IPA buffer, and then 3 × with chilled HPLC grade water. Then acetyl-lysine enriched peptides were eluted with 0.15% TFA for 10 min with gentle mixing. Peptide-containing supernatant was collected, and the elution was repeated with an additional 0.15% TFA, and the peptide-containing eluents were combined. The resulting acetylated lysine-enriched peptides were again purified using Sep-Pak C18 columns (Waters) and reconstituted in reverse phase LC buffer A for injection on the mass spectrometer.

### LC–MS/MS analysis of immunoprecipitated peptides and of global (total lysate) peptides

Acetylated peptides (1/3 of the eluted material following Sep-Pak, equivalent to approximately 2 µg of peptides) were loaded onto a self-packed 75 µm × 25 cm Picofrit emitter (New Objective) and eluted using a Dionex RSLCnano liquid chromatography system. The gradient consisted of a linear ramp from 3 to 42.5% buffer B (buffer A: 0.1% formic acid in water; buffer B: 0.1% formic acid in 80% acetonitrile) for a duration of 105 min. This was followed by a 20 min ramp to 60% buffer B, a 5 min ramp to 99% buffer B and 10 min flush at 99% buffer B. All flowrates were kept constant at 300 nl/min. Total lysate peptides eluted using a ramp from 3 to 50% buffer B over a 105 min gradient, followed by a 20 min ramp to 80% buffer B, a 5 min ramp to 99% buffer B and a 10 min flush at 99% buffer B. All peptide ions were collected by a Fusion Orbitrap mass spectrometer running at top speed mode with a cycle time of 5 s. Full scans (scan range from 400 to 1600 m/z) were collected at 120,000 resolution with a maximum injection time of 50 ms and an automatic gain control setting of 200,000. Higher energy collision dissociation (HCD) tandem mass spectra were collected in the ion trap with a maximum injection time of 35 ms and scan speed set to rapid. Collision energy was set to 30%; only ions with charge states between 2 and 7 were collected and dynamic exclusion was set to 20 s. Thermo raw data output is available on https://www.synapse.org/.

### MaxQuant searches for identification and label-free quantification (LFQ)

Raw files for total proteome and acetylome (acetyl lysine-enriched) were searched using MaxQuant’s integrated Andromeda search engine (version 1.5.2.8)^50^. Refseq v54 protein sequences (34,421 target sequences), were duplicated into a reverted (decoy) peptide database, searched, and used to control peptide and razor protein false discovery rate (FDR) at 1% within MaxQuant. Variable modifications of methionine oxidation (+ 15.9949 Da), and N-terminal acetylation (+ 42.0106 Da) plus fixed modification of cysteine carbamidomethylation (+ 57.0215 Da) were assigned. Lysine-specific acetylation (+ 42.0106 Da) was assigned as a variable modification for acetylated lysine-enriched peptide search. Tryptic peptides with up to 5 miscleavages were included for acetylome database search, and the default of 2 miscleavages was allowed for global proteins. A precursor mass tolerance of ± 20 ppm was applied prior to mass accuracy calibration and ± 4.5 ppm after internal MaxQuant calibration. Other search settings included a maximum peptide mass of 6000 Da, a minimum peptide length of 6 residues, 0.6 Da tolerance for low resolution MS/MS scans obtained in the linear ion trap. The false discovery rate (FDR) for peptide spectral matches, proteins, and site decoy fraction were all set to 1%. The label-free quantitation (LFQ) algorithm in MaxQuant was used for protein quantitation as previously described^51^. All raw files and MaxQuant search output data files are available on Synapse https://www.synapse.org/#!Synapse:syn26134616 (https://doi.org/10.7303/syn26134616).

### Data analysis

#### Imputation

Assumption of informative missingness was made for the imputation of missing LC–MS/MS protein LFQ (global protein measurements) or acetyl-lysine site-specific peptide precursor intensity values. Values were imputed from a random sampling of the Gaussian distribution with mean 1.8 standard deviations less than the population mean of all unimputed measurements and within ± 0.3 standard deviations from this mean, per parameters previously determined ideal for LFQ based studies^52^. As it is not reliable to impute global values if they are completely missing, only 1 out of 2 measurements was allowed missing in both the un-normalized raw values and in the global protein values for normalization. If both global measurements were never obtained for a particular protein (gene symbol), then no normalized values for change in knockdown or overexpression were obtained for any peptides from that protein. Such peptides from proteins unquantified in the global proteomes of total cell lysates are excluded from the normalized analysis.

#### Normalization

For each protein measured via global protein quantitative LFQ measurements, the LFQ ratio in the compared samples was used to adjust site-specific acetyl-lysine peptide intensity values in the same samples. Acetyl-lysine peptides were not considered when LFQ quantification of a matching protein was not available for any sample’s global protein sample measurements.

#### Differential expression (DEX)

DEX acetyl-lysine site-containing peptide intensities, with thresholds set at 50% change (1.5-fold of compared sample value) with SIRT2 knockdown or overexpression vs. paired control, were defined using quantitative site-level summary peptide intensities for all acetylated peptides found with FDR < 1% as defined by parameters of the MaxQuant search, after normalization to the respective level of the protein isoform from which they derived in the total cell lysate (global proteome LFQ intensity). Raw data for samples were numbered 1–8, where sample 1 was control-shRNA stably transfected, sample 3 was SIRT2 shRNA stably transfected, sample 5 was vector plasmid transfected together with acetyltransferases, and sample 7 was overexpressing SIRT2 together with acetyltransferases. Four additional even-numbered samples from same cellular background were part of the MaxQuant search for improving missingness due to MaxQuant’s borrowing of identifications across runs, but were not considered in downstream data analyses. Venn diagrams considered DEX hits from global protein level change-normalized values. Fifty percent change minimum, anti-correlated in the two opposing comparisons (SIRT2 knockdown/background and SIRT2 overexpression/background), was required for a global-protein-level-normalized acetyl-lysine site-specific peptide to be considered as a direct (negatively correlated with SIRT2 expression) or indirect (positively correlated with SIRT2 expression) (de)acetylation site downstream of SIRT2 activity.

#### False positive rate (FPR) test

To further test the reliability of our study, we also conducted FPR tests. For the current study, we consider the count of unlikely unidirectional decreases in both comparisons (−/−) for global protein abundance-normalized acetylation as false positives, and all remaining two-comparison changes beyond a sliding threshold (in either direction, but with at least one comparison to paired control increasing in acetylation; i.e. +/−, −/+, or +/+) as potential true (direct or indirect) positives influenced by differential SIRT2 activity. Our estimate of false positive rate (FPR, false positive count/potential SIRT2 activity-influenced true positive count) only considers proteins with at most 1 of 2 imputed values in both comparison arms of the study, therefore some proteins and sites identified as candidate direct targets of SIRT2 are not counted among the true positives for this purpose. FPR considering exact acetylated peptides changing in both knockdown and overexpression arms of the study was 9.0% (73/815), and considering protein-wide any site exceeding the threshold change in both study arms, FPR was 6.3% (32/504). For further validation, we considered whether “shuffled assumptions of change” using nonsensical pairs of the two controls and two experimental samples (scrambled assumption 1), or differences of nonsense differences (scrambled assumption 2) achieved a similar FPR. Neither scrambled assumption achieved below 25% FPR up to 50% minimum fold change across nonsense pairs’ calculated abundances as shown across the sliding threshold for site/peptide FPR (Supplementary Fig. S10a), and protein-level FPR (Supplementary Fig. S10b).

#### Ac-lysine enrichment by peptide spectral matches (PSMs)

To determine the overall specificity of the enrichment PSMs for acetylated lysine-containing peptides/total peptide PSMs for each of the four samples that underwent Ac-Lys enrichment with the Cell Signaling antibody prior to LC–MS/MS, we also conducted enrichment calculations from the alternate Proteome Discoverer (Thermo Scientific) software (PD) search for up to 3 Acetyl [K] per peptide, fully tryptic and 1% FDR enforced (high confidence peptide PSMs) (see Supplementary Table S5).

### Gene ontology analysis

Ontology enrichment in DEX lists of acetylated proteins—GO-Elite v1.2.5 (source code run on Python v2.7) and DAVID v6.8 (web interface) were used for ontology enrichment analysis. Fisher exact test was used for GO Elite and the background for both analyses was defined as all identified proteins in the experiment with the HCT116 cell line. To obtain an overview of the acetylated proteins after SIRT2 manipulation, we conducted a gene ontology (GO)-Elite functional analysis with all identified proteins (with 1.5-fold cutoff) based on their classification into biological processes (green bars), molecular functions (blue bars), and subcellular localization (red bars) GO categories as well as a custom analysis against the UCSD/Broad Institute molecular signatures database (MSigDB) C2 gene set list (https://www.gsea-msigdb.org/gsea/msigdb).

### KEGG analysis

To further understand the molecular processes involved with and enriched among identified hits, we also performed KEGG pathway analysis with the direct SIRT2 target protein gene products using the KEGG (Kyoto Encyclopedia of Genes and Genomes) database. Figure 3b shows the enriched KEGG signaling pathways with P value < 0.05.

### Motif identification and residues heat map

Sequence windows 134 peptides of SIRT2 direct targets were used. Motif-X was used via the Gygi lab website, with p < 0.001, a width of 11 residues, and up to 12 occurrences allowed, with the human proteome as background. For Motif-X, only the 134 31-residue pre-aligned modification site windows were input (centered at K ± 15 residues), and motifs were obtained.

Motif analysis was also performed on the MEME website. MEME was used to find overrepresented motifs with a high-frequency lysine (not forced to be central in the motif by the MEME algorithm, unlike Motif-X). For MEME, the peptide windows were converted to FASTA format and input as one peptide enriched with target motifs.

MEME was also used to generate a matrix of residue counts (position frequency matrix) for the 134 peptides’ 31-residue sequence windows centered on acetylated lysine, and then that matrix was used to perform a Fisher Exact Test and generate a -log_10_(p value) signed heatmap for each of the 20 amino acids at any position up to ± 10 residues from the central acetylated lysine (overuse beyond chance given by the frequency of residues in the human proteome is indicated by red, and underuse by green).

### MetaCore™ enrichment analysis and interaction network

The direct downstream gene list of SIRT2 (list of 184) was uploaded to MetaCore for enrichment analysis to determine Gene Ontology (GO) processes and molecular functions that were significantly based on P-value and ranked based – log10 (p-value). To elucidate the tightest communications and the most central connections, we adopted the direct interaction algorithm to develop the network, which is the most stringent algorithm and only allows the visualization of direct connectivity between root notes (proteins only from the provided list).

## Supplementary Information

Supplementary Figure S1.

Supplementary Table S1.

Supplementary Table S2.

Supplementary Table S3.

Supplementary Table S4.

Supplementary Table S5.
