# Supplementary material for: Quantitative proteomic analysis of the lysine acetylome reveals diverse SIRT2 substrates
Source: Sci Rep. 2022 Mar 9;12:3822. doi: 10.1038/s41598-022-06793-5 (PMC8907344; doi:10.1038/s41598-022-06793-5)
Supplement: Supplementary file 1 — Supplementary Figure S1. [file 41598_2022_6793_MOESM1_ESM.pdf]

a

b

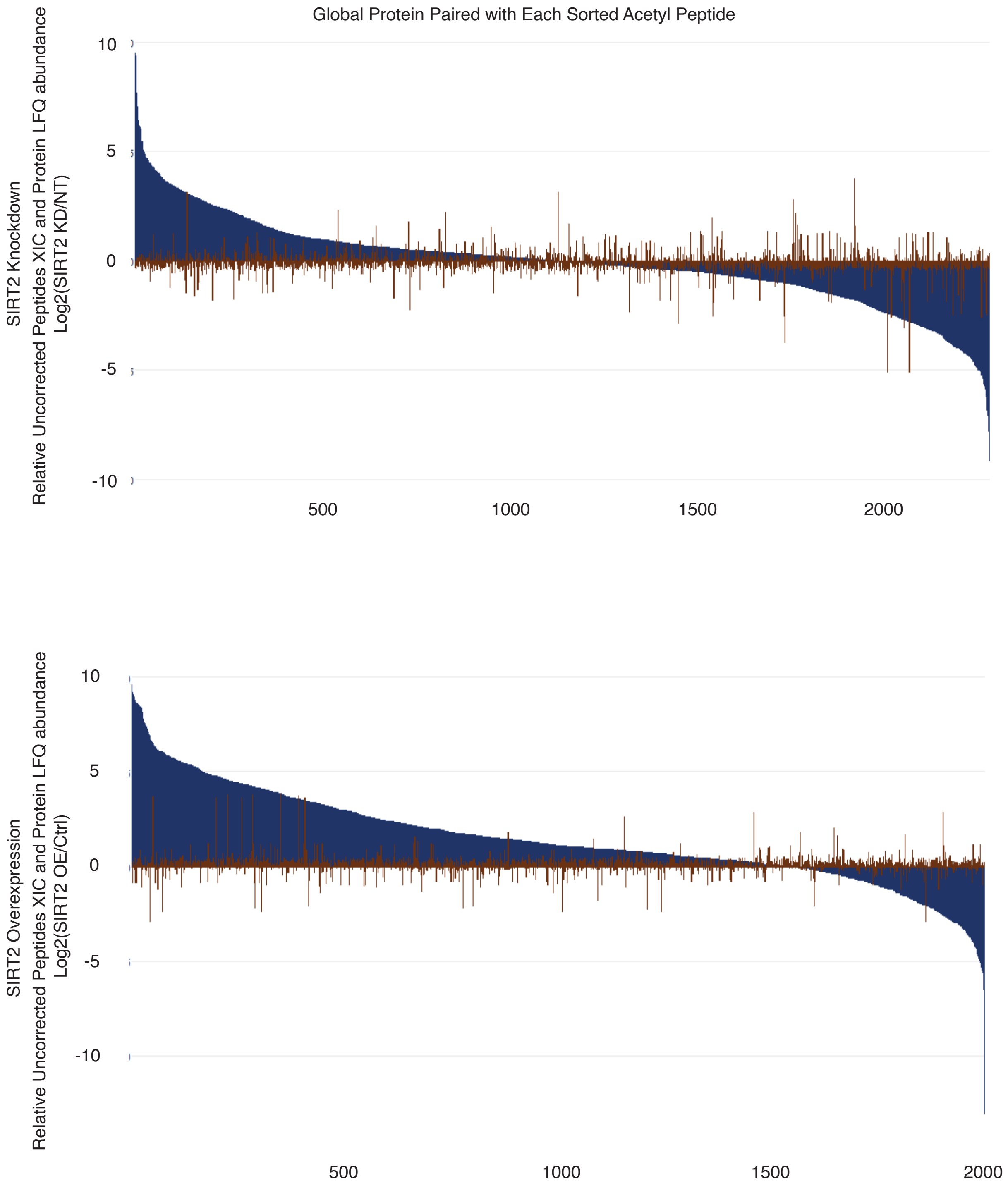

Supplementary Figure S1

a

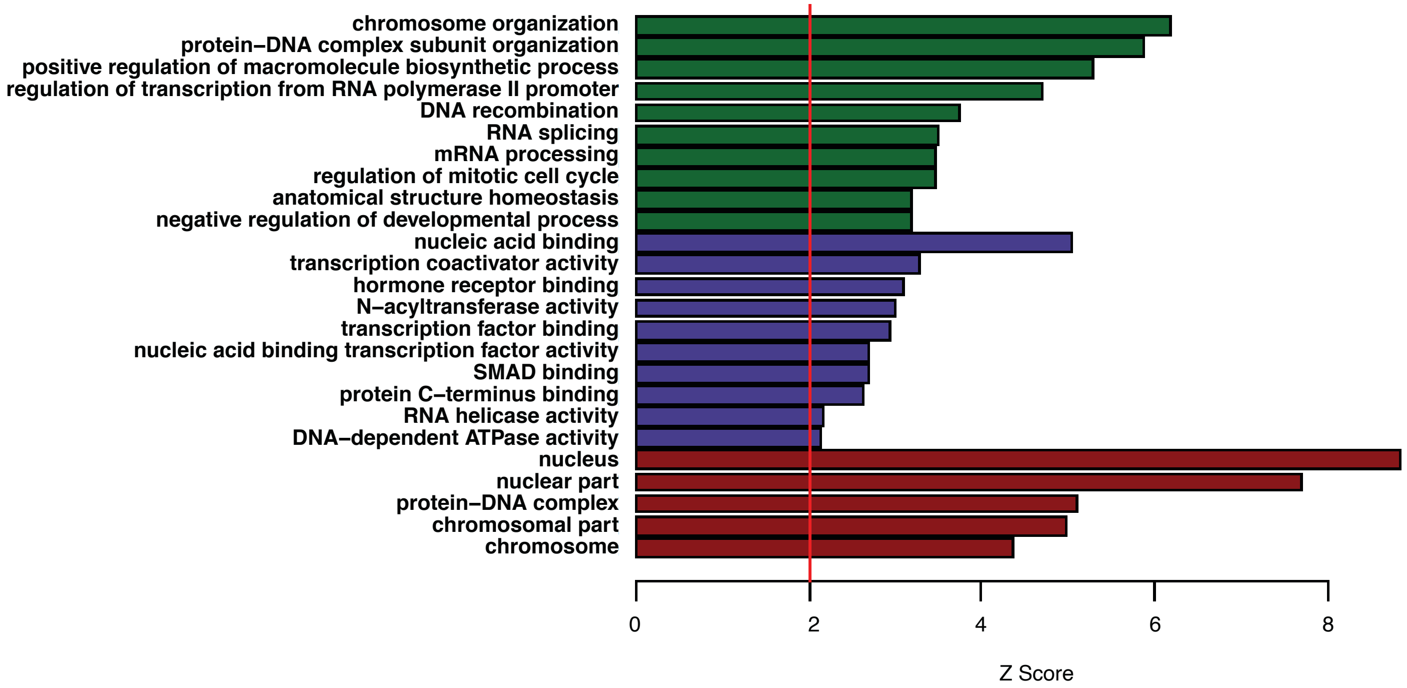

b

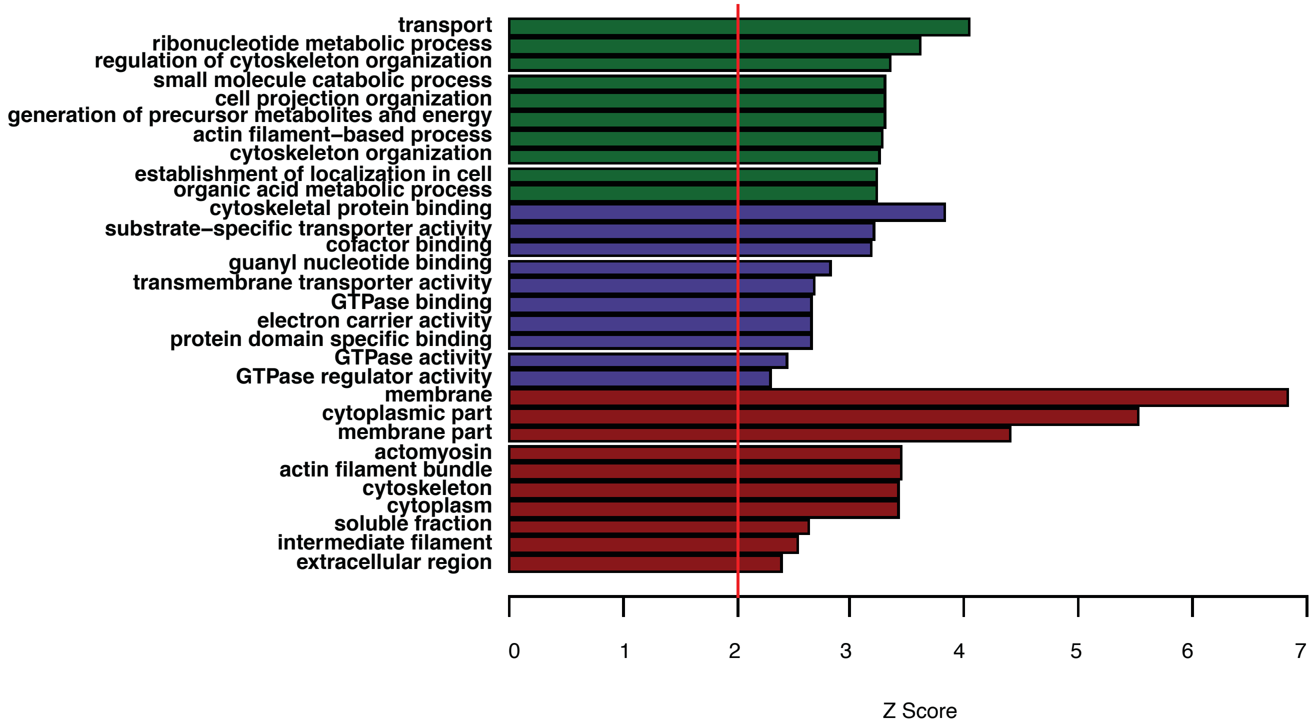

Supplementary Figure S2

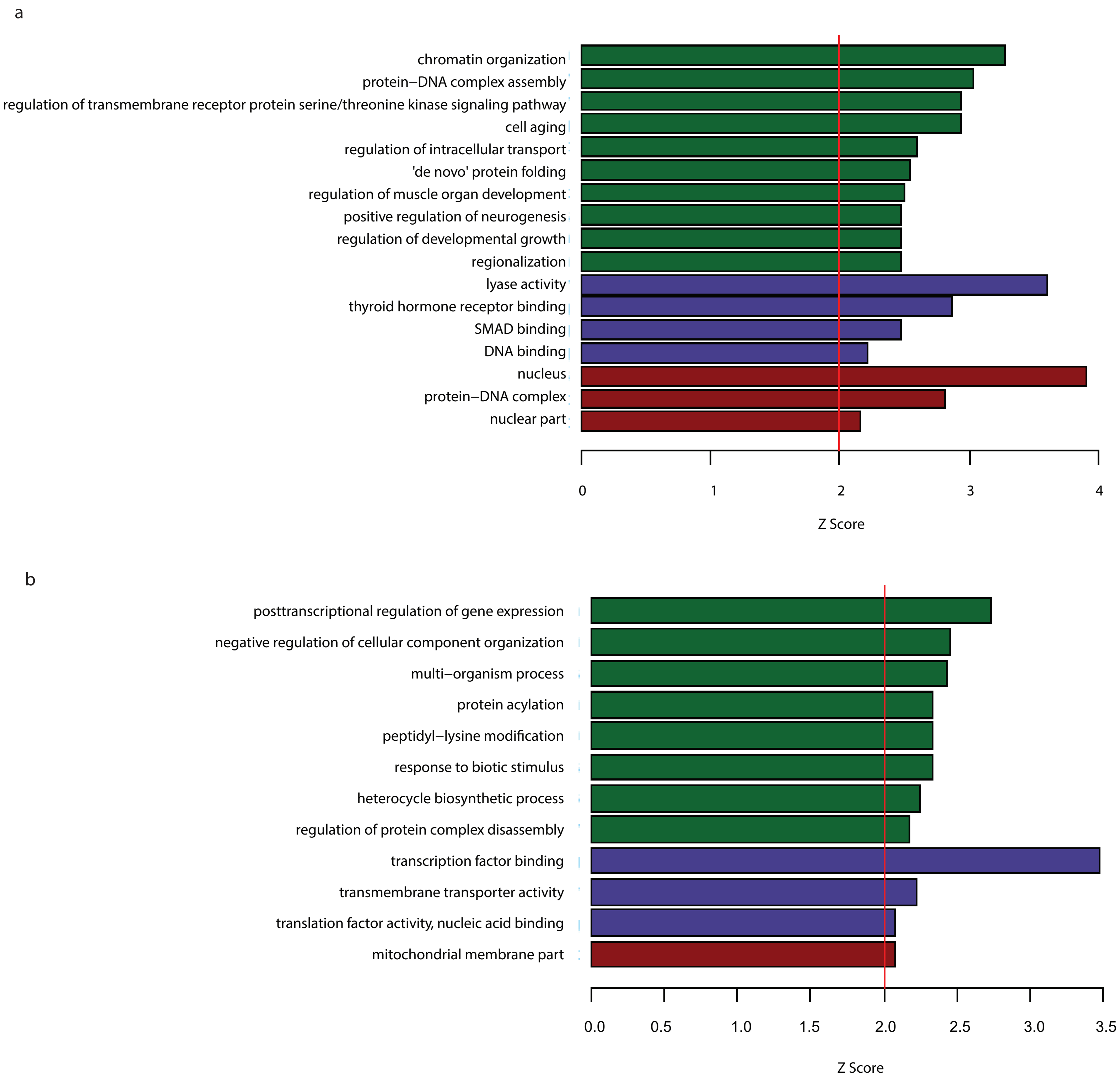

Supplementary Figure S3

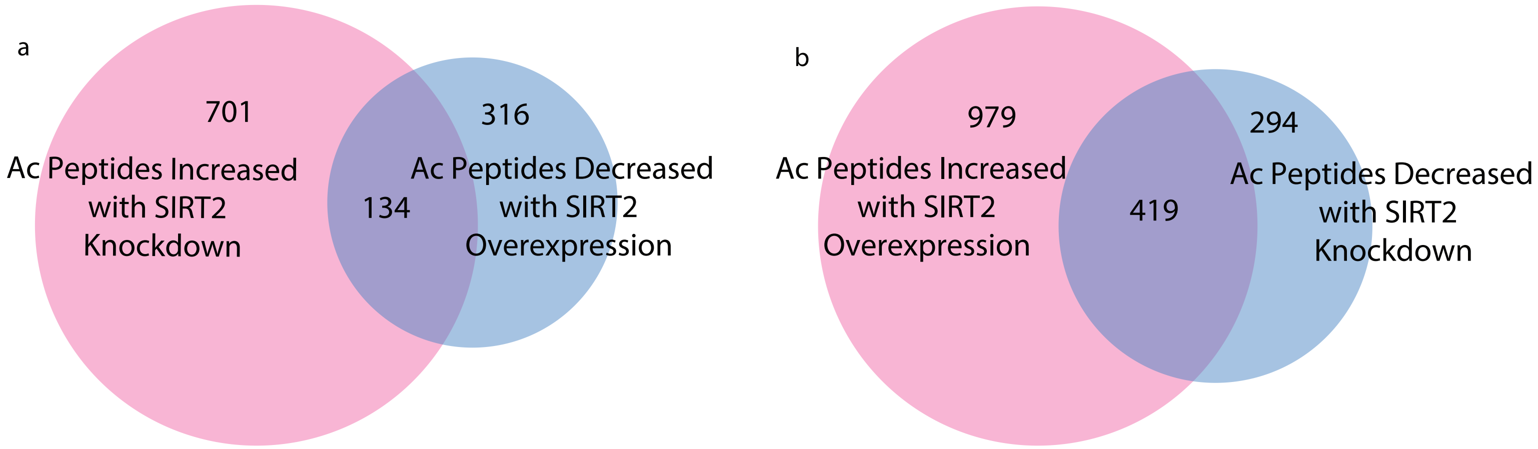

Supplementary Figure S4

a

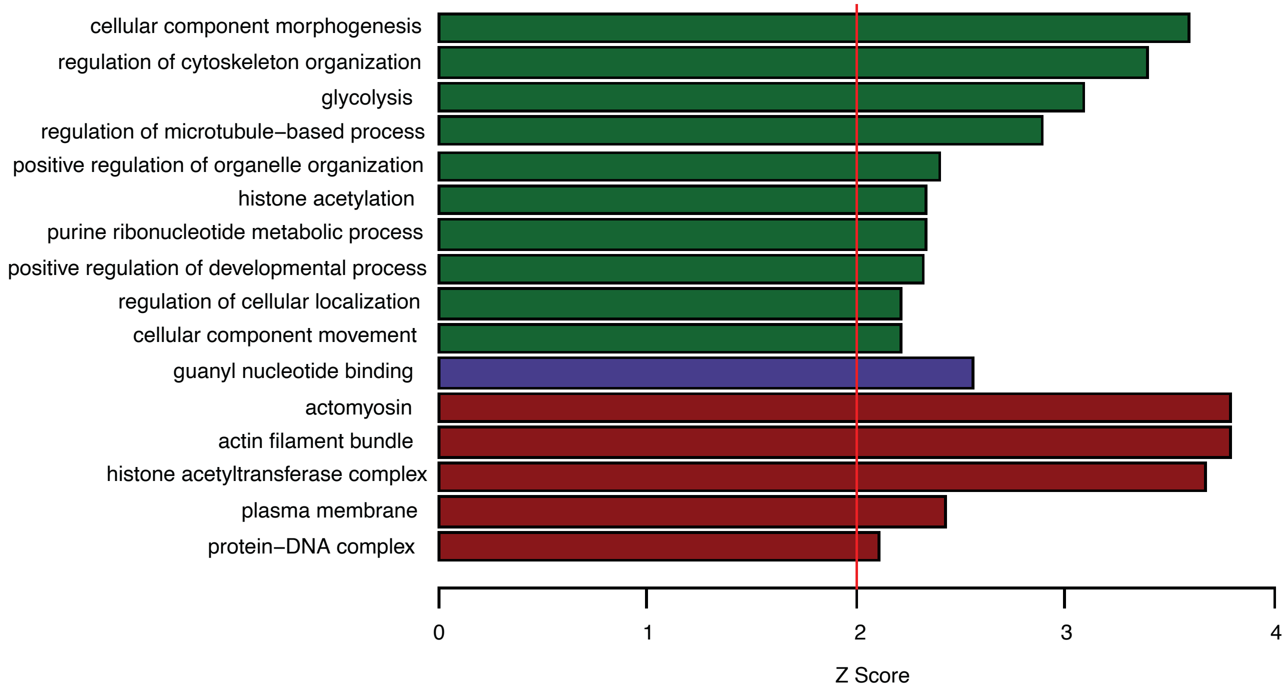

b

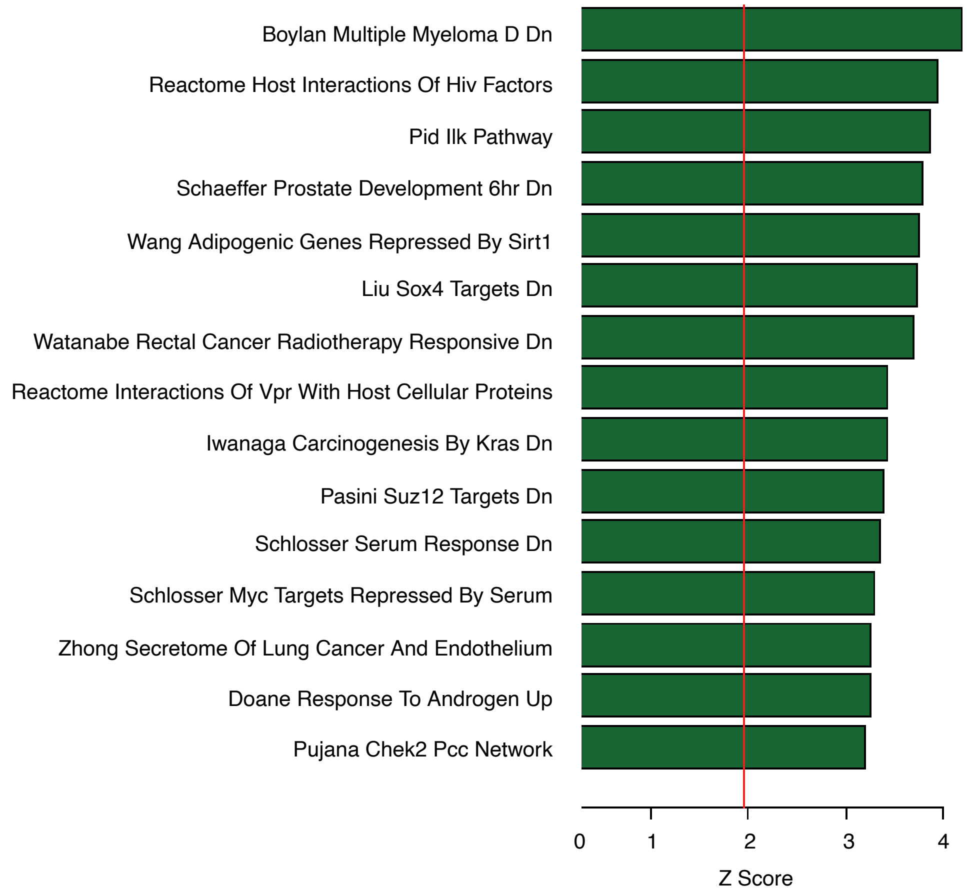

Supplementary Figure S5

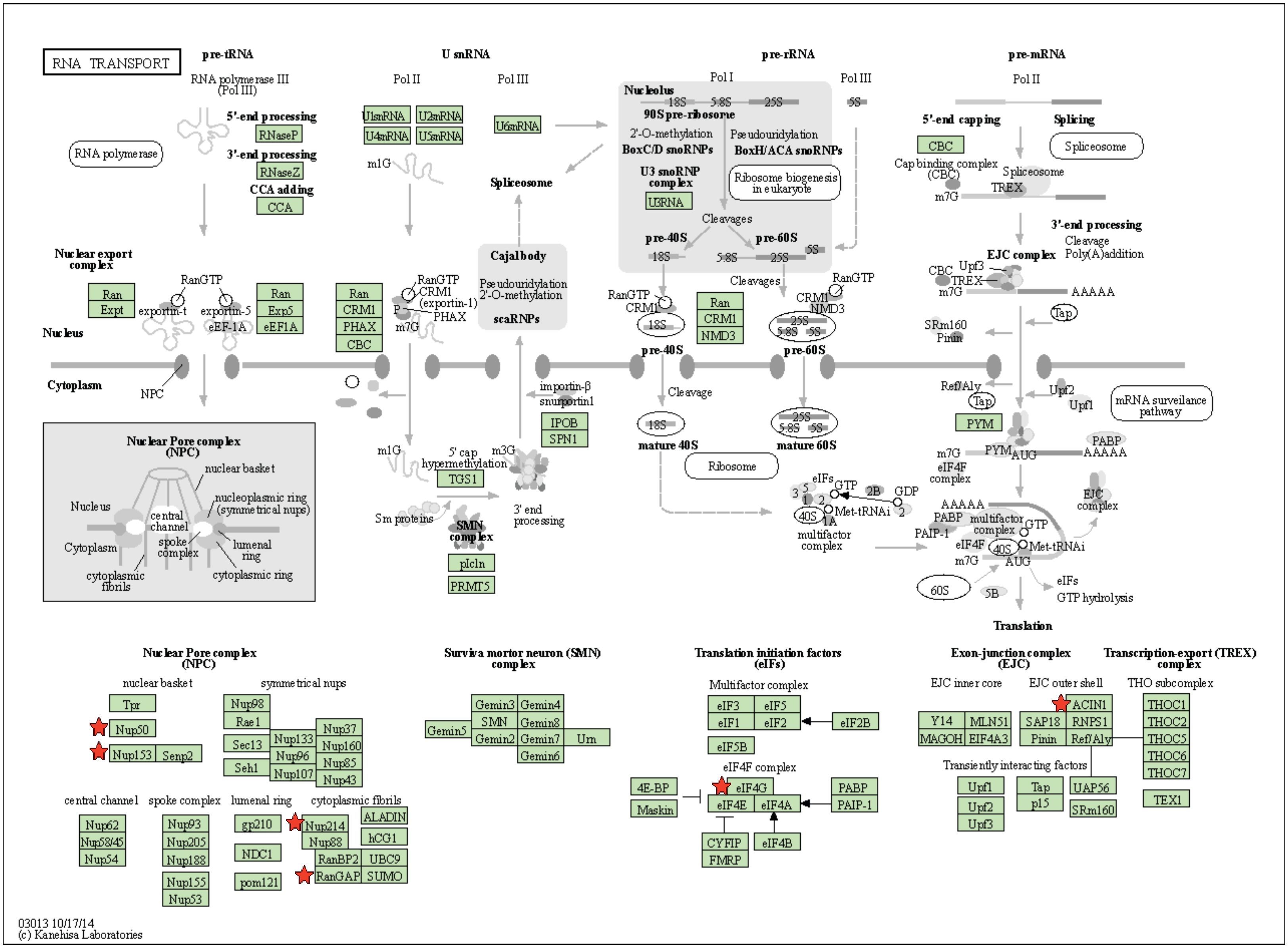

Supplementary Figure S6

a

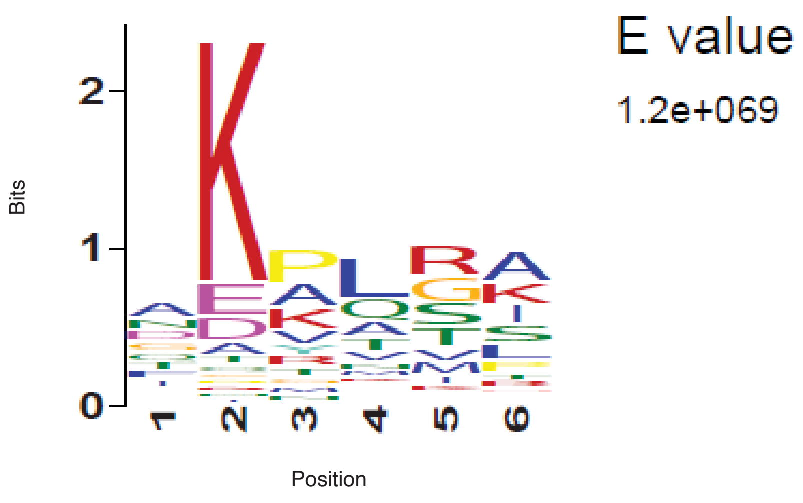

b

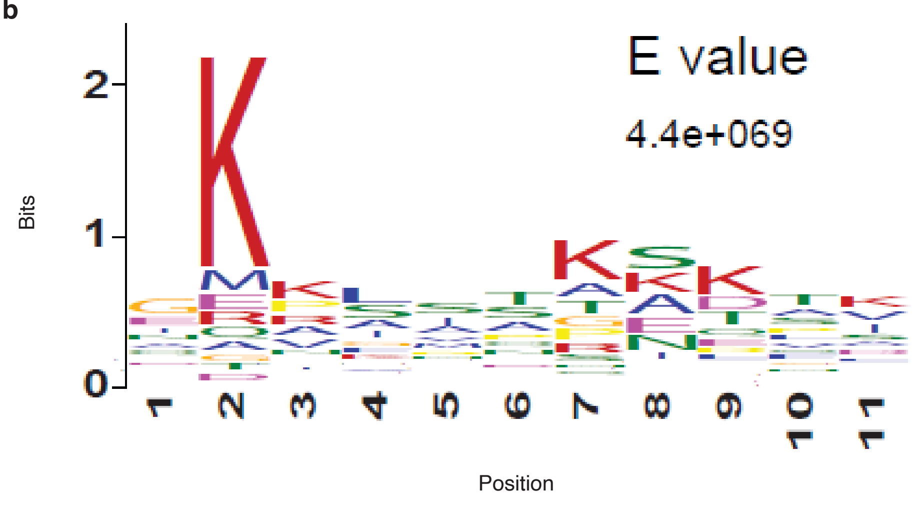

Supplementary Figure S7

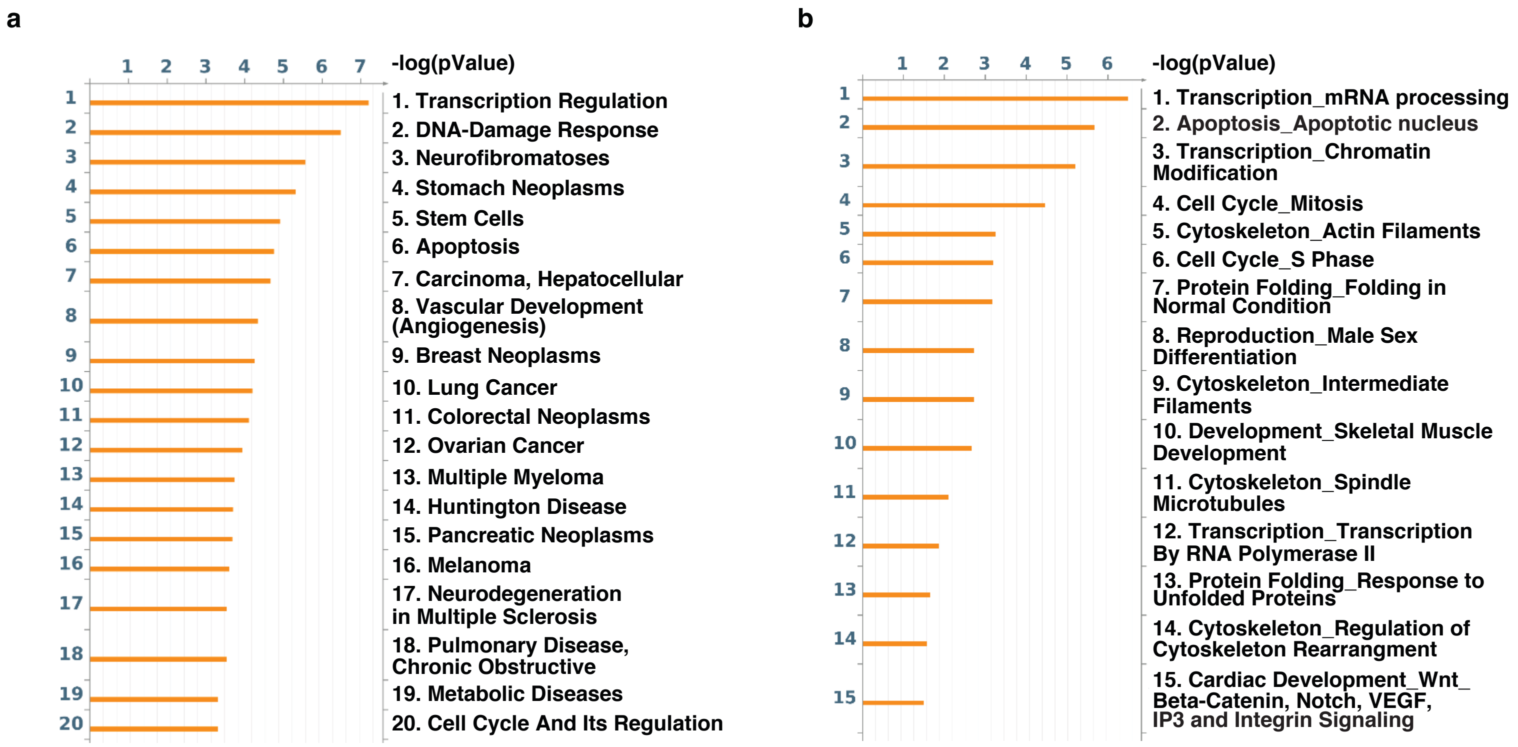

Supplementary Figure S8

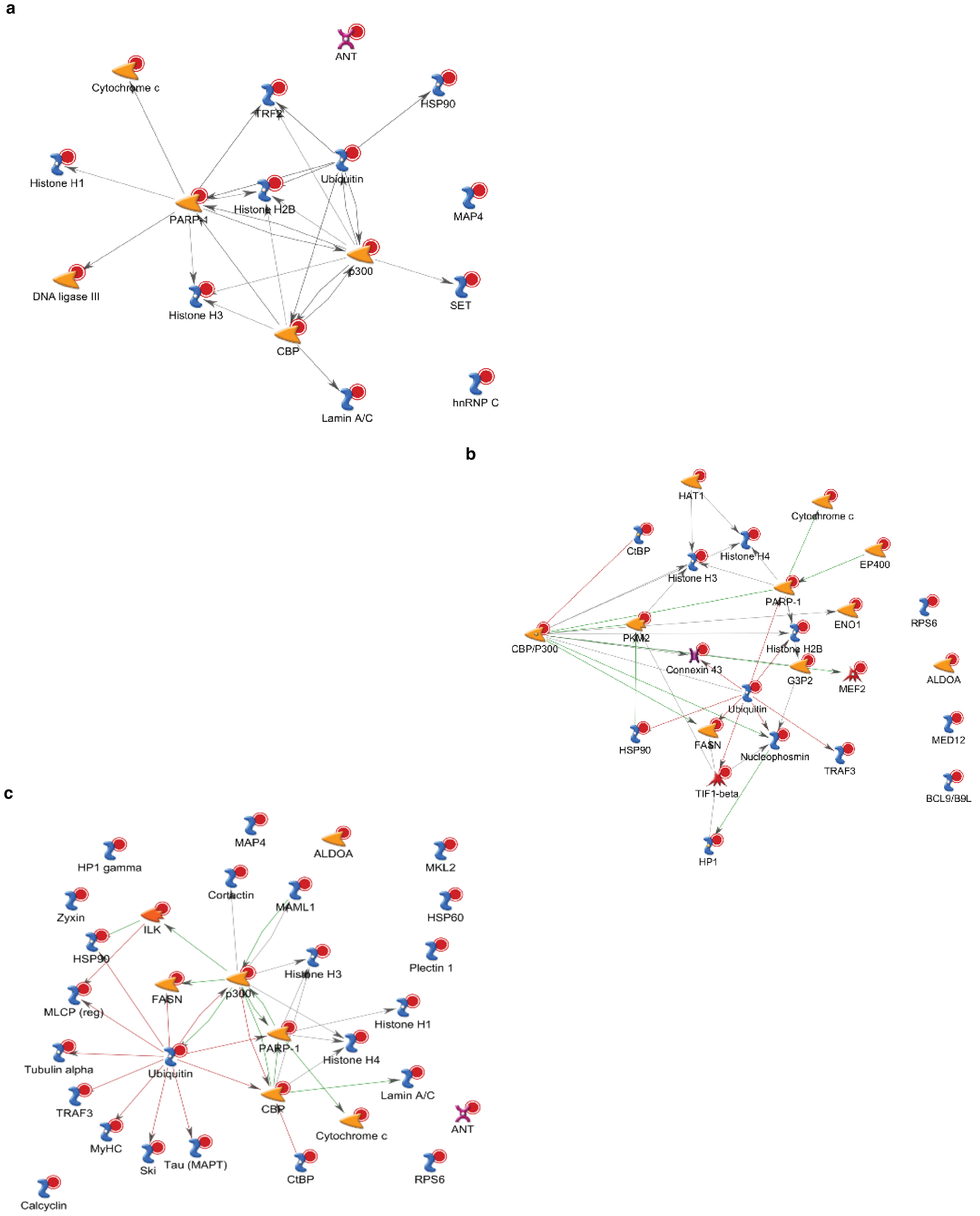

Supplementary Figure S9

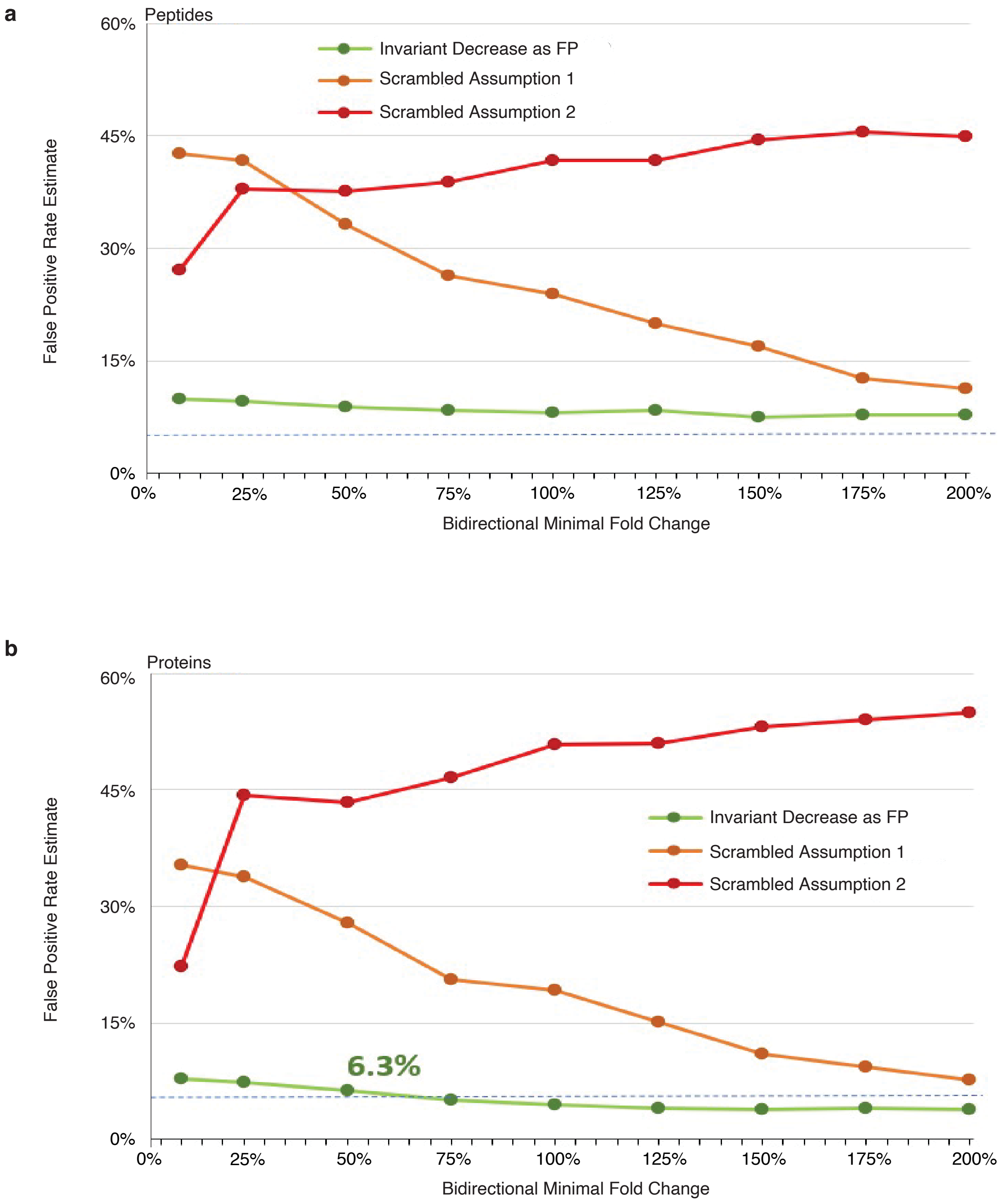

Supplementary Figure S10

Figure 1g

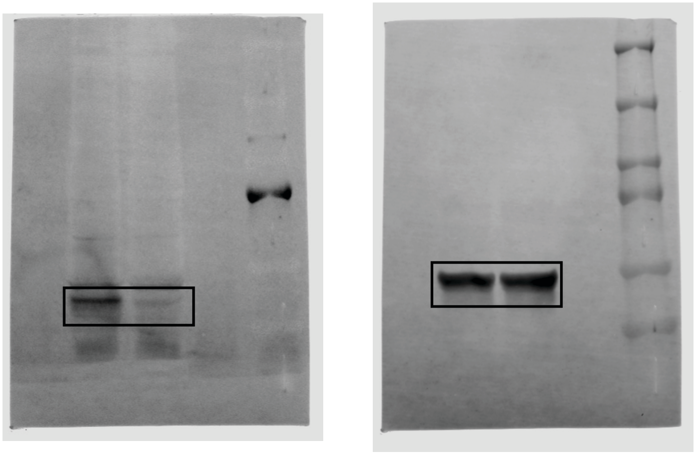

Figure 1h

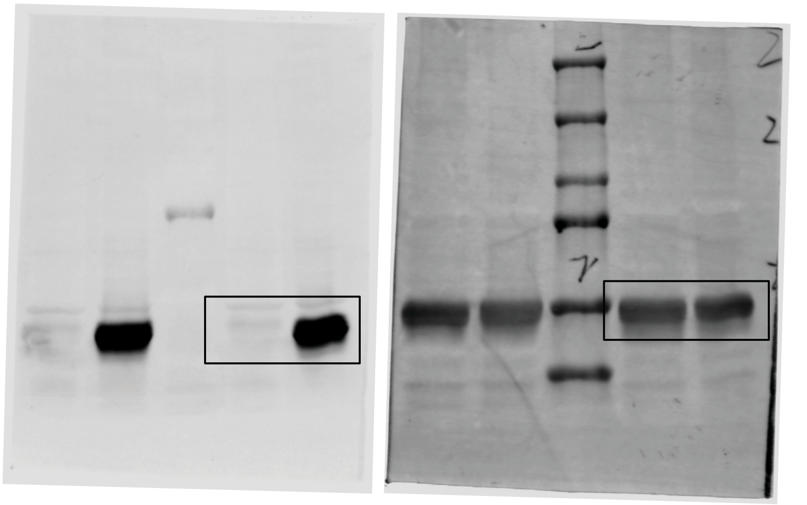

Figure S11a

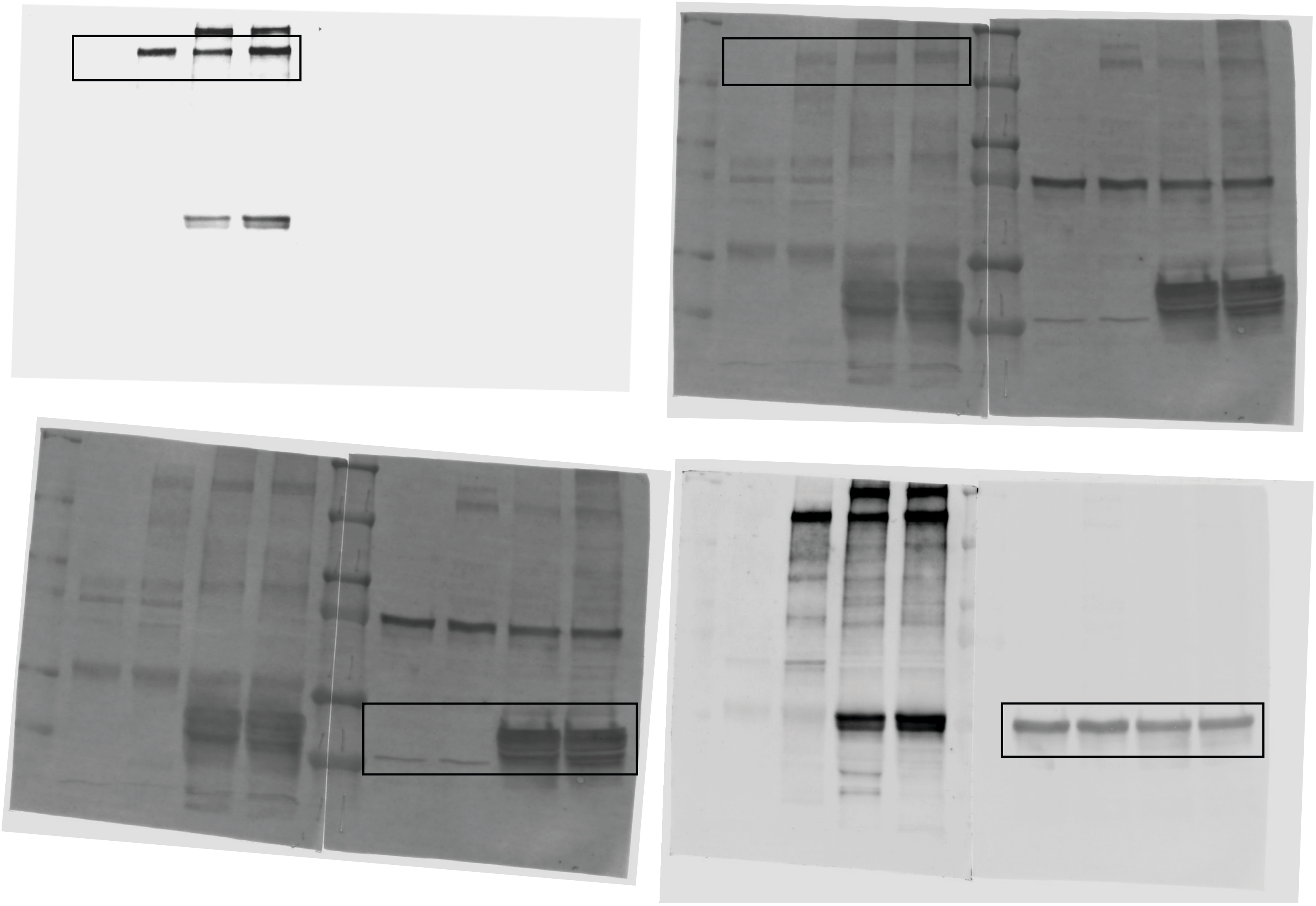

Supplementary Figure S11
